# Complete Genome Sequences of Three Important Methicillin-Resistant Clinical Isolates of *Staphylococcus pseudintermedius*

**DOI:** 10.1128/genomeA.01194-16

**Published:** 2016-10-20

**Authors:** Matthew C. Riley, Vincent Perreten, David A. Bemis, Stephen A. Kania

**Affiliations:** a Department of Biomedical and Diagnostic Sciences, Knoxville, Tennessee, USA; b Institute of Veterinary Bacteriology, Vetsuisse Faculty, University of Bern, Bern, Switzerland

## Abstract

We report the first complete genome sequences of three predominant clones (ST68, ST71, and ST84) of methicillin-resistant *Staphylococcus pseudintermedius* in North America. All strains were isolated from canine infections and have different SCC*mec* elements and antibiotic resistance gene patterns.

## GENOME ANNOUNCEMENT

*Staphylococcus pseudintermedius* is a Gram-positive opportunistic pathogen (1) that primarily causes infections in canines but is also relevant to human medicine (2–4), particularly with the worldwide expansion of methicillin-resistant clonal lineages (5–8). While a complete genome is publically available for a European methicillin-resistant *S. pseudintermedius* (MRSP) isolate (9), no complete MRSP genomes from dominant clonal lineages in North America are available. Here, we present the complete circular chromosomes of MRSP strains NA45, 081661, and 063228, which were isolated from canine infections in 2006 and 2008 and represent three dominant sequence types (ST) in North America, namely, ST84, ST71, and ST68, respectively (10).

All isolates were sequenced using Roche/454 (Roche Diagnostics, Switzerland), Illumina MiSeq (Illumina, Inc., USA), and Ion Torrent technologies (Thermo, Fisher Scientific, USA); isolates NA45 and 063228 were additionally sequenced using PacBio single-molecule real-time sequencing technology (Pacific Biosciences, USA). All genomes were mapped using the Argus Whole Genome Mapping System (Opgen, Inc., USA). *De novo* assemblies were individually produced and merged using Geneious version 9.1.3 (11) and CLC Genomics Workbench version 9.0 (https://www.qiagenbioinformatics.com). PacBio reads and whole-genome maps were used for scaffolding and genome closure (12). Automated annotation for strain 063228 was performed using the RAST server (http://rast.nmpdr.org), while the NCBI Prokaryote Genome Annotation Pipeline (http://www.ncbi.nlm.nih.gov/genome/annotation_prok) was used for NA45 and 081661.

The total number of high-quality reads for strain NA45 were 29,463 (PacBio), 583,182 (Illumina), 279,674 (Roche), and 4,660,374 (Ion Torrent), resulting in >250-fold overall coverage. The NA45 genome is 2,841,212 bp with a 37.3% GC content, 2,665 predicted coding sequences, and 78 predicted RNAs. High-quality reads for strain 081661 were 456,358 (Illumina), 129,593 (Roche), and 2,578,704 (Ion Torrent), resulting in >250-fold overall coverage. The 081661 genome is 2,731,109 bp with a 37.5% GC content, 2,610 predicted coding sequences, and 87 predicted RNAs. High-quality reads for strain 063228 were 24,585 (PacBio), 15,886,636 (Illumina), 113,288 (Roche), and 3,864,512 (Ion Torrent), resulting in >250-fold overall coverage. The 063228 genome is 2,766,566 bp with a 37.4% GC content, 2,734 predicted coding sequences, and 77 predicted RNAs.

The 081661 genome shared 99% identity over 96% of the published ST71 European isolate E140, with the major differences resulting from prophage composition (9). In addition to the methicillin resistance gene *mecA*, all strains contained the beta-lactamase gene *blaZ*, the kanamycin and streptomycin resistance genes *aph(3′)-III* and *ant(6)-Ia*. Strains 063228 and 081661 also harbor genes conferring resistance to gentamicin-kanamycin [*aac(6′)-Ie-aph(2′)-Ia*], macrolides-lincosamides-streptogramins B [*erm*(B)], while strain 063228 had additional lincosamide and tetracycline resistance genes *lnu*(A) and *tet*(M) (13). The methicillin resistance gene *mecA* was found on the staphylococcal cassette chromosome *mec* (SCC*mec*) SCC*mec*V in 063228, on SCC*mec*II-III in strain 081661, and on a novel SCC*mec* element in strain NA45. This 43,922-bp cassette has *mecA* integrated in the opposite direction compared to all other SCC*mec* elements (14) and contains the recombinase gene *ccrC6.*

The complete genomes of three strains belonging to three predominant clones causing infections in dogs in the United States permits further comparative genomic analyses and gives new insights into the molecular epidemiology and biological characteristics of *S. pseudintermedius*.

### Accession number(s).

These whole-genome projects have been deposited in DDBJ/ENA/GenBank under the accession numbers CP016072, CP016073, and CP015626. The versions described in this paper are the first versions, CP016072.1, CP016073.1, and CP015626.1.
